# Werner syndrome protein positively regulates XRCC4-like factor transcription

**DOI:** 10.3892/mmr.2014.2030

**Published:** 2014-03-10

**Authors:** DONGYUN LIU, XIAOLI DENG, CHONGZHEN YUAN, LIN CHEN, YUSHENG CONG, XINGZHI XU

**Affiliations:** 1 Beijing Key Laboratory of DNA Damage Response, College of Life Science, Capital Normal University, Beijing 100048, P.R. China; 2 Institute of Aging Research, Hangzhou Normal University School of Medicine, Hangzhou, Zhejiang 310036, P.R. China

**Keywords:** Werner syndrome, XLF, transcriptional regulation, cellular senescence

## Abstract

XRCC4-like factor (XLF) is involved in non-homologous end joining-mediated repair of DNA double-strand breaks (DSBs). Mutations in the *WRN* gene results in the development of Werner syndrome (WS), a rare autosomal recessive disorder characterized by premature ageing and genome instability. In the present study, it was identified that XLF protein levels were lower in *WRN*-deficient fibroblasts, compared with normal fibroblasts. Depletion of WRN in HeLa cells led to a decrease of *XLF* mRNA and its promoter activity. Chromatin immunoprecipitation assays demonstrated that WRN was associated with the *XLF* promoter. Depletion of XLF in normal human fibroblasts increased the percentage of β-galactosidase (β-gal) staining-positive cells, indicating acceleration in cellular senescence. Taken together, the results suggest that XLF is a transcriptional target of WRN and may be involved in the regulation of cellular senescence.

## Introduction

Our genomic DNA is constantly attacked and damaged by normal intracellular metabolic processes and a number of environmental factors, including ultraviolet (UV) light, ionizing radiation (IR), radiomimetic drugs and reactive oxygen species (ROS) (1,2). DNA damage also occurs spontaneously during DNA replication (2). To cope with these lesions, eukaryotic cells have evolved the complex DNA damage response (DDR) machinery (2). DDR senses damaged DNA and activates the cell cycle checkpoint to halt cell cycle progression, allowing time for DNA repair. The DDR process also induces apoptosis or senescence when damaged DNA fails to be repaired (2). Deficiency in DDR leads to mutations, genomic instability and cellular senescence, an enabling characteristic associated with cancer (3).

Of the various forms of DNA damage, DNA double-strand breaks (DSBs) are the most lethal (1). There are two main pathways that are independently responsible for DSB repair in mammalian cells, namely non-homologous end-joining (NHEJ) and homologous recombination (HR) (2). NHEJ is the predominant pathway, which induces the direct ligation of two broken DNA ends together (1,4). NHEJ repair occurs during G0 and G1 phase of the cell cycle, when the extensive sequence homology for repair is absent (1).

The NHEJ pathway is initiated by the rapid association of the Ku70/Ku80 heterodimer to the broken DNA ends (2). Ku70/Ku80 then recruits the catalytic subunit of the DNA-dependent protein kinase (DNA-PKcs) to form a DNA-PK complex (5). This kinase complex phosphorylates the nuclease Artemis to facilitate the initial processing of ends, and provides protection of the ends required for the following DNA ligation by another complex containing DNA ligase IV, XRCC4 and XRCC4-like factor (XLF; also called Cernunnos or NHEJ1) (2,6,7). XLF is structurally similar to XRCC4 and physically interacts with XRCC4. It is recruited to the DSB site in a Ku-dependent manner at an early stage of NHEJ (8,9) and facilitates XRCC4-mediated joining of blunt ends and several types of mismatched ends, that are non-complementary or partially complementary.

Deficiency in DDR pathways, including the NHEJ pathway, lead to premature ageing (10). Ku70, Ku80 or double knockout mice exhibit a series of age-related phenotypes, shortened life span and accumulation of chromosomal instability (11). Increased spontaneous translocations have also been identified in *XLF*-deficient murine embryonic stem cells (12). However, the link between XLF and ageing remains to be established.

Werner syndrome (WS) is a rare autosomal recessive progeroid syndrome characterized by the premature onset of multiple age-related disorders, including atherosclerosis, cancer predisposition and premature ageing. The *WRN* gene, when mutated, causes WS. The majority of the known *WRN* mutations are nonsense, producing truncated proteins lacking the nuclear localization signal (13). The WRN protein possesses helicase (14) and exonuclease (15) activities, and is important in multiple DNA metabolism pathways including DNA repair, recombination, replication and telomere maintenance (16). During the process of NHEJ, WRN is physically and functionally associated with, and regulated by, the major NHEJ factors, including the Ku70/Ku80 heterodimer, DNA-PKcs and the DNA ligase IV/XRCC4 complex, to optimize DNA end-processing (17–21).

WRN also functions in DNA transcription. It promotes RNA polymerase I-dependent transcription of ribosomal RNA (22) and is important in RNA polymerase II (RNA pol II)-dependent transcription (23). Transcription alterations have been identified in human fibroblasts from WS patients (24) and in the cells with RNAi-based short-term knockdown of *WRN* (25). The efficiency of RNA pol II transcription is reduced by ~50% in *WRN*-deficient cells compared with that in normal cells. The gene expression profiles in those cells resemble that of fibroblasts derived from old donor patients (24,25). The transcription alterations in WS are specific to sets of certain genes involved in different biological pathways, including DNA repair, DNA replication and cell cycle control (25), and may thus contribute to the development of the WS phenotype.

In the present study, it was identified that XLF was positively modulated by WRN and involved in the regulation of cellular senescence.

## Materials and methods

### Cell lines, siRNA oligos and antibodies

The human osteosarcoma cell line U2OS, human normal fibroblast cell line WI38, human normal fibroblast cell line GM00637 and human *WRN*-deficient fibroblast cell line AG11395 were purchased from the American Type Culture Collection (Manassas, VA, USA). The cell lines were cultured in DMEM medium with 10% fetal bovine serum (FBS; Hyclone Laboratories, Inc., Logan, UT, USA) and grown at 37°C in the presence of 5% CO_2_.

All predesigned siRNA oligonucleotide duplexes (OnTarget plus option) directed against human *XLF* or *WRN* (si-XLF or si-WRN) were purchased from Dharmacon, Inc. (Lafayette, CO, USA). The forward sequences of individual siRNA oligonucleotide duplexes were as follows, for si1-XLF: GCA UUA CAG UGC CAA GTG A dTdT; si2-XLF: CGC UGA UUC GAG AUC GAU UGA dTdT; si1-WRN: CUG UAU CUU CGG GCA CCA A dTdT and i2-WRN: UGA AGA GCA AGU UAC UUG C dTdT. The forward sequence of control siRNA oligonucleotide duplex (si-CTR) was CGU ACG CGG AAU ACU UCG A dTdT.

Mouse monoclonal antibody against β-actin (clone AC15) was purchased from Sigma (St. Louis, MO, USA). Antibodies against XLF (BL3263) and WRN (BL1309) were purchased from the Bethyl Laboratories (Montgomery, TX, USA). Peroxidase-conjugated secondary antibodies were from Jackson ImmunoResearch (West Grove, PA, USA).

### Cell growth assay

WI38 cells were transfected with si-XLF or si-CTR with RNAiMAX. Cells were trypsinized 24 h following transfection and transferred into 6-well plates (1×10^4^ cells/well). The cell number was counted every day for 5 days, with triplicated wells being used at each time point.

### Senescence-associated β-galactosidase (SA-β-gal) staining

WI38 cells at passage 39 were infected with a control siRNA (si-CTR) or XLF-specific siRNAs (si1-XLF or si2-XLF). Transfectants were cultured for 10 days and processed for SA-β-gal staining as described by Dimri *et al* (26). Briefly, the cells were washed with PBS and fixed with 0.5% glutaraldehyde in PBS for 5 min at room temperature. Following washing with PBS, the cells were incubated with a freshly prepared staining solution [1 mg/ml 5-bromo-4-chloro-3-indolyl-β-D-galactopyranoside (X-gal), 40 mmol/l citric acid/sodium phosphate (pH 6.0), 5 mmol/l potassium ferrocyanide, 5 mmol/l potassium ferricyanide, 15 mmol/l NaCl and 2 mmol/l MgCl_2_] at 37°C for 16 h.

### Western blot analysis

Cell lysates were prepared in 0.5% NP-40 lysis buffer [50 mmol/l Tris (pH 8.0), 250 mmol/l NaCl, 5 mmol/l EDTA, 0.5% NP40] containing protease inhibitor cocktail (Roche Diagnostics, Indianapolis, IN, USA). The protein concentration was determined using an DC assay kit (Bio-Rad, Hercules, CA, USA). Equal amounts of proteins were resolved on 4–18% gradient SDS-PAGE gels and transferred onto nitrocellulose membranes (Bio-Rad). The blots on nitrocellulose were blocked with 5% non-fat milk in PBST (PBS with 0.05% Tween-20) and were sequentially incubated with primary antibodies as indicated and horseradish peroxidase-conjugated secondary antibodies in 5% non-fat milk in PBST. Blots were washed with PBST following each incubation. The immunoreactive bands were visualized by Amersham Biosciences ECL reagents ((Piscataway, NJ, USA) following the manufacturer’s instructions.

### Transfections and dual luciferase reporter assays

siRNA oligonucleotide duplex at a final concentration of 40 μM, was transfected into U2OS cells twice with a 24 h interval using oligofectamine (Invitrogen Life Technologies, Carlsbad, CA, USA) according to the manufacturer’s instructions. Transfectants were used for further experiments 24 h following the secondary transfection. For dual luciferase reporter assays, on the day prior to siRNA transfection, 5×10^4^ cells were seeded into each well of 24-well plates. Following the secondary siRNA transfection (4 h), a firefly luciferase reporter construct under the control of the *XLF* promoter (1 μg) and a Renilla luciferase reporter construct under the control of the TK promoter for normalization of transfection efficiency (10 ng) were co-transfected into cells in triplicate using FuGENE6 (Roche Diagnostics) at a ratio of 1 μg of plasmid to 3 μl of FuGENE6. Luciferase activity was determined with the dual luciferase assay system (Promega Corporation, Madison, WI) 48 h following the first siRNA transfection. Relative light units were determined using a luminometer (microtiter plate luminometer). Experiments were performed at least three times independently and each combination was tested in triplicate wells.

### Reverse-transcription PCR and real-time reverse-transcription PCR

Total RNA was extracted from U2OS cells using the TRIzol reagent (Invitrogen Life Technologies). Reverse transcription (RT)-PCR was performed using the Access Quick RT-PCR system (Promega Corporation) essentially according to the manufacturer’s instructions. Real-time PCR was performed using the QuantiTect SYBR-Green PCR kit (Qiagen, Hilden, Germany) and the iQ5 thermal cycler (Bio-Rad). Primers used for amplification of GAPDH were GAPDH: sense, TGG TAT CGT GGA AGG ACT CA and antisense, CCA GAT GAG GCA GGG ATG AT. Primers used for amplification of XLF were XLF: sense, GAG TCC ACG GGT ACT TCA GG and antisense, GGG CCT GTC AAC ATC AAC TT.

### Chromatin immunoprecipitation (ChIP)

ChIP assays were performed as previously described (27). The ChIP-enriched DNA was amplified by PCR with primer pairs that are specific for the *XLF* promoter sequence. *XLF* promoter first fragment corresponds to the position from −596 to −337, primers used were forward: GGT ACC GAA GGG ATA ATG AAT TCT GAT TGG GGA CAG and reverse: GGA TCC TCC GAC CTC ATC CTT TAC CTC TCC TGC TTC. *XLF* promoter second fragment corresponds to the position from −360 to −90, primers used were forward: GGT ACC GGA GAG GTA AAG GAT GAG GTC GGA CTA TG and reverse: GGA TCC GGC TAG TAG AAG GGT AGT GGC GCG TCT TG. *XLF* promoter third fragment corresponds to the position from −81 to +169, primers used were forward: GGT ACC GGC CTC TCC TCC ACT TAC CCT GGC CAC TG and reverse: GGA TCC GAC TCG AAC GCG ATT CCA CCT ACC GTC AG.

## Results and Discussion

### XLF is a transcriptional target of WRN

**Figure 1:**
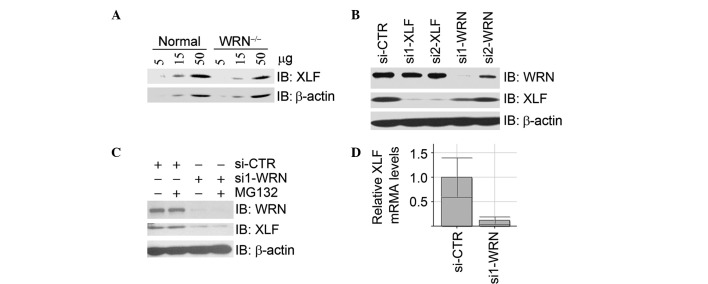
WRN regulates mRNA levels of XLF. (A) XLF protein levels decreased in *WRN*-deficient fibroblasts. Whole cell lysates were prepared from human fibroblasts GM00637G (normal) and AG11395A (*WRN* deficient) and subjected to immunoblotting analysis for XLF and β-actin. (B) Inhibition of WRN expression by siRNA resulted in a decrease of XLF protein levels. U2OS cells were transfected with si-CTR, XLF siRNA (si1-XLF or si2-XLF) or WRN siRNA (si1-WRN or si2-WRN). Total cell lysates were harvested 48 h following transfection and subjected to immunoblotting analysis with the antibodies indicated. (C) MG132 treatment did not restore the decrease of XLF protein levels caused by the depletion of WRN. U2OS cells were treated as described in (B) except that MG132 was added to cells 4 h prior to harvest. (D) Inhibition of *WRN* expression led to a decrease of XLF mRNA. U2OS cells were treated as described in (B). Total RNA was extracted and subjected to real-time RT-PCR assays for XLF mRNA. XLF, XRCC4-like factor; si-CTR, control siRNA; RT-PCR, reverse transcription PCR.

A candidate gene approach was employed to identify DDR factors in the human fibroblast cell line AG11395A, which was originally isolated from a *WRN* patient bearing a nonsense mutation in the *WRN* gene. It was identified that the endogenous XLF protein level was lower in AG11395A cell line than in the control fibroblast cell line GM00637G (Fig. 1A). In U2OS cells, inhibition of *WRN* expression by two independent siRNA oligos resulted in a decrease of XLF protein levels (Fig. 1B), which were not restored by treatment with the proteasome inhibitor MG132 (Fig. 1C). Real-time RT-PCR assays demonstrated that the mRNA levels of XLF were significantly decreased upon depletion of *WRN* by siRNA (Fig. 1D). Taken together, these data suggest that WRN is a positive regulator of XLF at the transcriptional level.

### WRN positively regulates the XLF promoter activity

**Figure 2:**
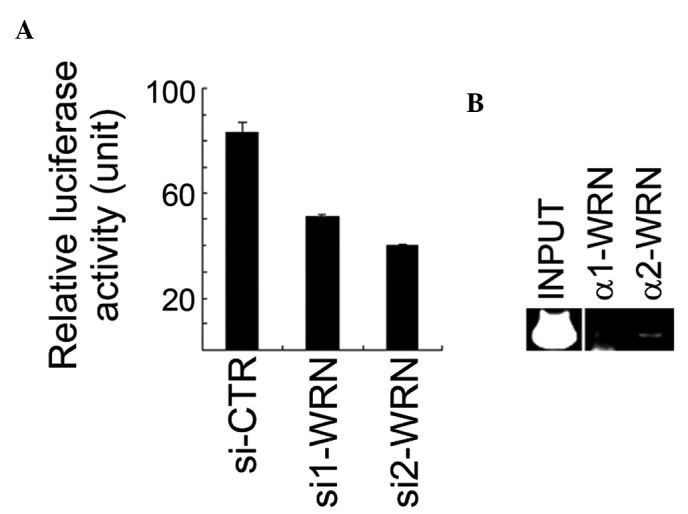
WRN positively regulates the *XLF* promoter activity in U2OS cells. (A) Inhibition of *WRN* expression decreased the *XLF* promoter activity. The putative XLF promoter containing a sequence from −596 to +169, relative to the transcription start site of *XLF*, was subcloned into the PGL3-basic luciferase vector, and the resulting reporter construct was transfected into *WRN*-depleted U2OS cells. Triplicate samples were analyzed. (B) WRN bound the *XLF* promoter region. ChIP assays were performed using two different anti-WRN antibodies. ChIP with α1-WRN antibody did not yield any PCR product, which also served as a negative control. The PCR product corresponds to the position from −360 to −90 of the promoter relative to the transcription start site. XLF, XRCC4-like factor; ChIP, chromatin immunoprecipitation; si-CTR, control siRNA.

To determine if WRN regulates the promoter activity of *XLF*, the putative XLF promoter region from −596 to +169 (relative to the putative transcription start site described in the Ensembl protein-coding gene ENSG00000187736) was cloned into the pGL3 basic luciferase reporter vector. The resulting pGL3-XLFpr was identified to have pronounced luciferase reporter activity in U2OS cells (data not shown). It was identified that the *XLF* promoter activity was significantly downregulated when *WRN* expression was inhibited by siRNA in U2OS cells (Fig. 2A). The antibodies against WRN were used for ChIP on cross-linked chromatin fragments prepared from U2OS cells. The ChIP-enriched DNA was subjected to PCR analysis using three pairs of primers for amplification of three fragments within the *XLF* promoter region. The data revealed that the second fragment (from −360 to −90) of the *XLF* promoter region was detected in the anti-WRN immunoprecipitates (Fig. 2B). These results suggest that WRN resides on the *XLF* promoter region, positively regulating its activity.

### Depletion of XLF promotes cellular senescence in normal human fibroblasts

**Figure 3:**
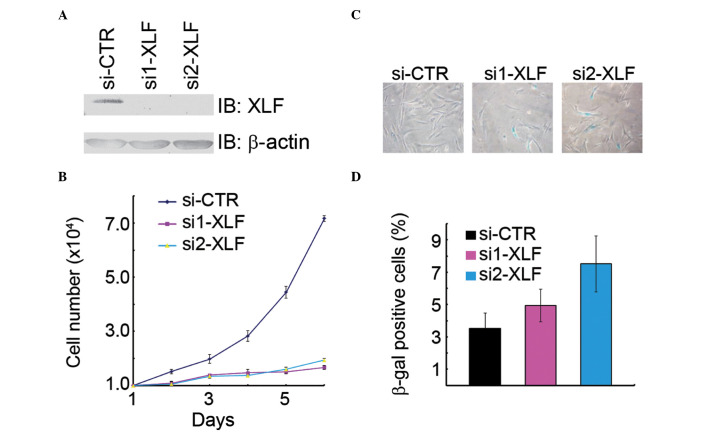
XLF suppresses cellular senescence. (A) Inhibition of XLF expression in WI38 cells. WI38 cells at passage 39 PD were transfected with si-CTR or si-XLF. Total cell lysates were extracted 48 h following transfection and subjected to immunoblotting analysis with the antibodies indicated. (B) Depletion of XLF inhibited cell proliferation. XLF expression was inhibited by transfection with si-XLF as described in (A) and cell numbers were determined every 24 h. Six duplicate samples were analyzed at each time point. (C and D) Inhibition of XLF expression accelerated cellular senescence. XLF-depleted WI38 cells were assayed for β-gal. Representative images of stained cells are shown in (C) and quantitative data in (D). PD, population doubling; XLF, XRCC4-like factor; β-gal, β-galactosidase; si-CTR, control siRNA.

The prominent biological function of XLF is to repair DSBs by NHEJ, while the central effect of *WRN* deficiency is premature cellular senescence. It is well known that defects in NHEJ-mediated DSB repair contribute to cellular senescence. Therefore, we hypothesized that defects in *XLF* would lead to premature senescence. Indeed, inhibition of *XLF* expression by transfection with two independent siRNA oligos resulted in a decrease of cell growth in the normal human fibroblasts WI38 (Fig. 3A and B), while an increase in the percentage of β-gal-positive cells as compared with the mock transfectants (Fig. 3C). Therefore, it was concluded that XLF is critical in promoting cell proliferation and suppressing cellular senescence.

WRN is known to physically and functionally interact with the DNA-PK kinase complex and the XRCC4/Ligase IV complex, two key complexes involved in NHEJ. The DNA-PK kinase complex has been demonstrated to phosphorylate WRN *in vivo* and *in vitro*, and this phosphorylation inhibits the WRN helicase and exonuclease activities. By contrast, the interaction between XRCC4 and WRN, stimulates WRN’s exonuclease activity, enabling it to serve as a DNA end-processing factor during NHEJ. In the present study, the results reveal a novel mechanism of the functional interplay between WRN and the NHEJ process, in which WRN positively regulates XLF at the transcription level. Furthermore, these data provide evidence for the first time, to the best of our knowledge, of the functional link of XLF to cellular senescence.

## References

[1] Jackson SP (2002). Sensing and repairing DNA double-strand breaks. Carcinogenesis.

[2] Ciccia A, Elledge SJ (2010). The DNA damage response: making it safe to play with knives. Mol Cell.

[3] Hanahan D, Weinberg RA (2011). Hallmarks of cancer: the next generation. Cell.

[4] Lees-Miller SP, Meek K (2003). Repair of DNA double strand breaks by non-homologous end joining. Biochimie.

[5] Mladenov E, Iliakis G (2011). Induction and repair of DNA double strand breaks: the increasing spectrum of non-homologous end joining pathways. Mutat Res.

[6] Buck D, Malivert L, de Chasseval R (2006). Cernunnos, a novel nonhomologous end-joining factor, is mutated in human immunodeficiency with microcephaly. Cell.

[7] Ahnesorg P, Smith P, Jackson SP (2006). XLF interacts with the XRCC4-DNA ligase IV complex to promote DNA nonhomologous end-joining. Cell.

[8] Yano K, Morotomi-Yano K, Wang SY (2008). Ku recruits XLF to DNA double-strand breaks. EMBO Rep.

[9] Yano K, Chen DJ (2008). Live cell imaging of XLF and XRCC4 reveals a novel view of protein assembly in the non-homologous end-joining pathway. Cell Cycle.

[10] Hasty P (2008). Is NHEJ a tumor suppressor or an aging suppressor?. Cell Cycle.

[11] Li H, Vogel H, Holcomb VB, Gu Y, Hasty P (2007). Deletion of Ku70, Ku80, or both causes early aging without substantially increased cancer. Mol Cell Biol.

[12] Zha S, Alt FW, Cheng HL, Brush JW, Li G (2007). Defective DNA repair and increased genomic instability in Cernunnos-XLF-deficient murine ES cells. Proc Natl Acad Sci USA.

[13] Martin GM, Austad SN, Johnson TE (1996). Genetic analysis of ageing: role of oxidative damage and environmental stresses. Nat Genet.

[14] Gray MD, Shen JC, Kamath-Loeb AS (1997). The Werner syndrome protein is a DNA helicase. Nat Genet.

[15] Huang S, Li B, Gray MD, Oshima J, Mian IS, Campisi J (1998). The premature ageing syndrome protein, WRN, is a 3′-->5′ exonuclease. Nat Genet.

[16] Luo J (2010). WRN protein and Werner syndrome. N Am J Med Sci (Boston).

[17] Li B, Comai L (2000). Functional interaction between Ku and the werner syndrome protein in DNA end processing. J Biol Chem.

[18] Yannone SM, Roy S, Chan DW (2001). Werner syndrome protein is regulated and phosphorylated by DNA-dependent protein kinase. J Biol Chem.

[19] Li B, Comai L (2002). Displacement of DNA-PKcs from DNA ends by the Werner syndrome protein. Nucleic Acids Res.

[20] Otsuki M, Seki M, Kawabe Y (2007). WRN counteracts the NHEJ pathway upon camptothecin exposure. Biochem Biophys Res Commun.

[21] Kusumoto R, Dawut L, Marchetti C (2008). Werner protein cooperates with the XRCC4-DNA ligase IV complex in end-processing. Biochemistry.

[22] Shiratori M, Suzuki T, Itoh C, Goto M, Furuichi Y, Matsumoto T (2002). WRN helicase accelerates the transcription of ribosomal RNA as a component of an RNA polymerase I-associated complex. Oncogene.

[23] Balajee AS, Machwe A, May A (1999). The Werner syndrome protein is involved in RNA polymerase II transcription. Mol Biol Cell.

[24] Kyng KJ, May A, Kølvraa S, Bohr VA (2003). Gene expression profiling in Werner syndrome closely resembles that of normal aging. Proc Natl Acad Sci USA.

[25] Turaga RV, Paquet ER, Sild M (2009). The Werner syndrome protein affects the expression of genes involved in adipogenesis and inflammation in addition to cell cycle and DNA damage responses. Cell Cycle.

[26] Dimri GP, Lee X, Basile G (1995). A biomarker that identifies senescent human cells in culture and in aging skin in vivo. Proc Natl Acad Sci USA.

[27] Rauch T, Zhong X, Pfeifer GP, Xu X (2005). 53BP1 is a positive regulator of the BRCA1 promoter. Cell Cycle.

